# Dr. Dewey’s Enterprise

**Published:** 1876-10

**Authors:** 


### DR. DEWEY'S ENTERPRISE.

Our old friend, Dr. George A. Dewey, announces in this issue of the Journal his inauguration of a Purchasing Agency for physicians, invalids, heads of hospitals, and all who have occasion to buy medicines, surgical instruments, or remedial appliances of any kind. This strikes us as a good idea, because the world is full of sophisticated drugs and bad medicines, and it is not an easy thing for physicians, even, to select the best. Of course the layman is completely at the mercy of quacks and adulterators, and medicines which are bad enough at best, are rendered doubly base by the additions which are calculated only to cheapen, but which in reality render them wholly untrustworthy, if not absolutely dangerous.

Now, we are not great admirers of drugs—although educated to trust in them implicitly—because we know of very valuable agencies outside the pharmacopia—food, air, exercise, sunlight, water, the absence of fret, a serene temper ; but when drugs are to be used, let us at least know that they are genuine. Dr. Dewey is an educated physician, a fine writer, a chemist of no ordinary skill, and in every respect a trustworthy gentleman. We have no doubt that friends at a distance, doctors as well as patients, will be glad to employ so competent a gentleman in procuring medicinal substances and the like.

